# Flower Diversification Across “Pollinator Climates”: Sensory Aspects of Corolla Color Evolution in the Florally Diverse South American Genus *Jaborosa* (Solanaceae)

**DOI:** 10.3389/fpls.2020.601975

**Published:** 2020-12-07

**Authors:** Marcela Moré, Ana C. Ibañez, M. Eugenia Drewniak, Andrea A. Cocucci, Robert A. Raguso

**Affiliations:** ^1^Laboratorio de Ecología Evolutiva y Biología Floral, Instituto Multidisciplinario de Biología Vegetal, CONICET-Universidad Nacional de Córdoba, Córdoba, Argentina; ^2^Department of Neurobiology and Behavior, Cornell University, Ithaca, NY, United States

**Keywords:** floral evolution, floral reflectance, pollinator vision, pollinators’ color space, hawkmoth pollination, saprophilous fly pollination, pollinator climates

## Abstract

Flower phenotype may diverge within plant lineages when moving across “pollinator climates” (geographic differences in pollinator abundance or preference). Here we explored the potential importance of pollinators as drivers of floral color diversification in the nightshade genus *Jaborosa*, taking into account color perception capabilities of the actual pollinators (nocturnal hawkmoths vs. saprophilous flies) under a geographic perspective. We analyzed the association between transitions across environments and perceptual color axes using comparative methods. Our results revealed two major evolutionary themes in *Jaborosa*: (1) a “warm subtropical sphingophilous clade” composed of three hawkmoth-pollinated species found in humid lowland habitats, with large white flowers that clustered together in the visual space of a model hawkmoth (*Manduca sexta*) and a “cool-temperate brood-deceptive clade” composed of largely fly-pollinated species with small dark flowers found at high altitudes (Andes) or latitudes (Patagonian Steppe), that clustered together in the visual space of a model blowfly (*Lucilia* sp.) and a syrphid fly (*Eristalis tenax*). Our findings suggest that the ability of plants to colonize newly formed environments during Andean orogeny and the ecological changes that followed were concomitant with transitions in flower color as perceived by different pollinator functional groups. Our findings suggest that habitat and pollination mode are inextricably linked in the history of this South American plant lineage.

## Introduction

In a seminal study on pollinator-driven flower diversification, Grant and Grant (1965) coined the expression “pollinator climate” to explain potential selective forces driving floral specialization on different kinds of pollinators (or to self-pollination) across their geographical ranges. Concerning selective pressures, Grant and Grant (1965) stated that latitudinal or altitudinal differences in pollinator abundance-pollinator climate-are to floral phenotype as environmental factors are to vegetative phenotype. Consequently, as any given abiotic environment may have several covarying dimensions such as temperature, wind and humidity that define a multidimensional selective context, pollinators likewise impose, through their own integrated phenotypes of anatomical and behavioral peculiarities, a multidimensional biotic selective context. More recently, the factors contributing to pollinator climate have been formalized as dimensions of pollinator niche space (Johnson, 2010; Phillips et al., 2020). The pollinator climate/niche approach has been used to formulate and test predictions for local pollinator specialization among populations of specific plants (e.g., *Erica plukenetii* in the western Cape region of South Africa; Van Der Niet et al., 2014) as well as pollination themes in plant communities from different regions (e.g., short vs. long-tongued pollinator guilds; Johnson et al., 2017). One factor that unites the emerging concept structure of pollinator climate/niche with the formalization of pollinator “functional groups” (Fenster et al., 2004) is the expectation that the sensory biases and capabilities of pollinators constitute fundamental dimensions of pollinator climate/niche, leading to consistent behavioral preferences for certain kinds of flowers, the “attraction” component of Grant’s body of work on floral isolation (Grant, 1994; Hodges et al., 2004).

An important aspect in considering the role of pollinators as drivers of floral diversification is the context in which diversification is promoted. When considering the geographic perspective, it is possible to distinguish whether floral divergence is or is not associated with changes in the local pollinator context (see Ellis and Johnson, 2009). For example, floral diversification may occur when plants disperse to geographical areas where the pollinator fauna is depauperate in relation to the source area (Streisfeld and Kohn, 2007). Southern South America is one of the few places in the world where subtropical lowlands lie in close geographical proximity to glaciated high mountain ranges, providing the rare opportunity to assess the influence of markedly contrasting eco-geographic domains in floral diversification mediated by pollinator climates. The topographical and environmental changes that occurred during the Andean orogeny have been crucial both in the origin of new biomes and in the concurrent diversification of Andean lineages (Chaves et al., 2011; Luebert and Weigend, 2014; Strelin et al., 2017). Studies on the paleobotany and geology of South America suggest that up to half of the Central Andes uplift (i.e., a rise from 2000 m to the present 4000 m) has taken place in the last 10 Ma. The combination of maximum altitudes and globally cooling temperatures date the origins of alpine Andean vegetation to ca. 3–5 Ma, making it the youngest natural ecosystem of South America (Graham, 2009). The formation of alpine and steppe habitats with cold desert climate about 3–5 Ma bp challenged resident organisms with a completely new context to which to adapt. For plants, the pollination environment changed dramatically. The emergence of these new habitats represented an unprecedented adaptive challenge with regard to pollination climate. Flies, comprising between 67 and 77% of the pollinator fauna diversity, are the dominant pollen vectors both in alpine (see Arroyo et al., 1982; Devoto et al., 2005) and high latitude environments (Elberling and Olesen, 1999). In contrast, other animals are dominant pollinators in the sub-alpine shrub zone and the subtropical lowland areas where bees (Devoto et al., 2005; Chacoff et al., 2012) or nocturnal hawkmoths (Sazatornil et al., 2016), respectively, are relatively more important pollinators. Consequently, we would expect that plants evolving in an area that has markedly changed in pollinator climate would be under selection to adjust their flower phenotypes accordingly.

Floral color is an important visual signal for pollinator attraction. Because different animal lineages possess distinctive color-sensitive visual receptors and cognitive capabilities, the way each pollinator group (e.g., bats, hummingbirds, flies, lepidopterans, bees) perceives color is unique (Briscoe and Chittka, 2001; Schaefer and Ruxton, 2011). Considering the hypothesis that flower color signals evolved to match the visual systems of pollinators (Chittka and Menzel, 1992), it is expected that flowers present corolla colors that exploit their pollinators’ visual capability and preferences (De Ibarra and Vorobyev, 2009; Shrestha et al., 2013; Paine et al., 2019). However, pollinator attraction is not the only mechanism driving the evolution of flower color. Several case studies have shown that corolla color diversification can result from pollinator shifts (=attracting different functional groups; review by Rausher, 2008), from interspecific competition among pollinators of the same functional group (=promoting floral constancy; Muchhala et al., 2014) or from avoiding antagonistic flower visitors (=reducing florivory or larceny; Irwin et al., 2003; Gutierrez De Camargo et al., 2019; Chen et al., 2020).

Floral traits in most plant species have the potential to adapt to new conditions under changing selective pollination environments (Kay et al., 2005; Whittall and Hodges, 2007). Such shifts in pollination systems may occur quite rapidly through novel floral mutations. For example, a single allelic substitution is sufficient to explain flower color change in monkey flowers, representing an adaptive shift between bumblebees and hummingbirds as pollinators (Bradshaw and Shemske, 2003). Flower color is a labile trait and several studies have shown that color change is spurred by adaptive pollinator shifts, i.e., a given color evolved in concert with pollination by a specific functional group of pollinators. Some of the most common transitions of pollination systems are from bee to moth, bee to bird and bird to moth (review by Rosas-Guerrero et al., 2014). However, only one case of transition between brood site deception by saprophilous fly and nocturnal hawkmoth -and vice-versa- has been reported so far in a complex of milkweed species in Japan (Yamashiro et al., 2008). Flowers that use brood-site deception to attract saprophilous flies as pollinators are well known for their unusual floral morphology, visual and olfactory display (review by Urru et al., 2011). These two modes of pollination are so utterly different (brood site deception of saprophilous flies vs. honest –nectar rewarding– pollination by hawkmoths) as to require, in theory, many changes in floral phenotype to integrate the sensory cues that attract pollinators with the functional morphology necessary to effect pollen transfer (Phillips et al., 2020). In this study, we explore a putative moth-fly pollinator shift from the standpoint of flower color, in the South American genus *Jaborosa*.

The genus *Jaborosa* Juss. (Solanaceae) is endemic to southern South America and exhibits strong inter-specific variation in floral traits across geographic domains. Such variation ranges from night-blooming white fragrant flowers with very long corolla tubes (Vesprini and Galetto, 2000) that produce abundant nectar, to diurnal, dark pigmented, unpleasant smelling flowers with shallow corollas that produce little to no nectar (Moré et al., 2013, 2019). Two utterly different pollination modes have been described associated with these extreme phenotypes: nectar-rewarding flowers pollinated by nocturnal hawkmoths and rewardless brood-site deceptive flowers pollinated by saprophilous flies, respectively. Geographical distribution of hawkmoth-pollinated species is restricted to humid subtropical lowlands, whereas carrion-fly pollinated species grow in semiarid regions located either at high altitudes in the Andes or at high latitudes in the Monte desert and the temperate Patagonian steppe of southernmost Argentina (Moré et al., 2015). Given the floral diversity and present geographic distribution of the genus *Jaborosa* as well as the geological history of the region now occupied by *Jaborosa*, we expect that flower evolution in *Jaborosa* reflects ancestral movement across contrasting pollinator climates. In particular, floral evolution should have been subjected to shifts along the perceptual dimension of the changing pollinator climates. Changes in flower signaling should bear on visual conspicuousness in terms of chromatic and achromatic contrast vis-à-vis the perceptual capabilities of the geographically and historically changing pollinators. Consequently, we first expect color conspicuousness to be greater in plant species with nocturnal flowers than in those with diurnal ones (see Kuenzinger et al., 2019). Second, we expect the timing of the shifts to be consistent with the geological events that created new pollination climates. Third, we expect that the phenotypic transitions across pollination climates occurred along perceptual dimensions of the changing pollinators. In the present study we use plant-phylogenetically informed and pollinator-visual modeling approaches to assess the evolution of color diversity within the genus *Jaborosa*, taking into account the color perception capabilities of the current pollinators serving as potential selective agents.

## Materials and Methods

### Study System

**FIGURE 1:**
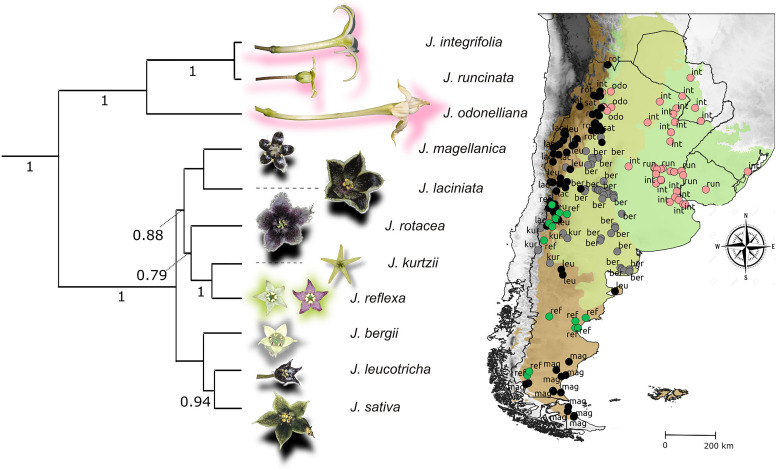
Study system. Flower coloration, phylogenetic relationships and geographical distribution of *Jaborosa* species. Color shading in the map shows the ecoregions classification proposed by Olson et al. (2001) as follows: herb green: lowlands; olive green: subalpine; brown: alpine (see Supplementary Table 2 for details). Colors of the circles show pollination mode: pink for nocturnal hawkmoths, black for saprophilous flies, green for generalized pollination by small insects, and gray for unknown pollinators, respectively. Numbers in the tree show branch support; only Bayesian posterior probabilities higher than 0.75 are shown.

The nightshade genus *Jaborosa* comprises 22 species that exhibit remarkable interspecific variation in floral traits (Figure 1). Previous phylogenetic analyses recovered two strongly supported clades with geographic affinities: a “subtropical lowland clade,” including the three species with sphingophilous (hawkmoth-pollinated) flowers inhabiting lowland areas, and an “andean clade,” including the remaining species, mainly distributed across the higher altitudes (Andes mountain range) and higher latitudes (temperate South America) (Moré et al., 2015). A phylogenetic hypothesis for *Jaborosa* was estimated using Bayesian inference from the four-gene plastid dataset collected from 30 taxa, including 19 *Jaborosa* species and 11 representatives of outgroup genera (Moré et al., 2015). The Bayesian analysis was performed using BEAST v.1.7.5 (Drummond et al., 2012) under a relaxed clock model with branch-specific rates following a lognormal distribution and a GTR+G model of nucleotide substitution with a randomly generated starting-tree topology and Yule speciation process. The topology shown here is a maximum clade credibility (MCC) tree pruned to show only the 11 taxa studied here using the phytools 0.7-20 package (Revell, 2012) of R software (R Core Team, 2020; Figure 1).

### Pollinators

Field studies were conducted in several localities of Argentina to characterize the pollinators of each species of *Jaborosa*. Mean observation time per species was 223 min, ranging from 60 to 630 min (Supplementary Table 1). Floral visitors were recorded during day and nighttime (whenever climatic conditions allowed) by direct observation, photography and video in the field. Representative specimens of insects visiting the flowers were captured using a hand-held net for later identification, with voucher specimens deposited at the Laboratorio de Ecología Evolutiva y Biología Floral (IMBIV, Córdoba, Argentina). Additionally, stigmas were inspected for the presence of lepidopteran scales in the field using a hand lens (20×), as evidence of recent visitation. Nocturnal sampling of hawkmoth individuals was carried out by means of light traps in two localities (Termas de Reyes and El Fuerte populations, Jujuy Province; see Moré et al., 2006 for details) within the range of *Jaborosa integrifolia*. Hawkmoths were collected for identification of pollen loads in the laboratory as described by Schlumpberger et al. (2009).

### Spectra Acquisition and Processing

We measured corolla reflectance across 11 *Jaborosa* species representing the full spectrum of floral coloration in the genus (Figure 1 and Supplementary Table 1). Measurements were taken from fresh flowers (4–11 individuals per species and 1–3 flowers per individual when available) and leaves (1–3 per species) as background against which flowers are displayed. We used SpectraSuite software (Ocean Insights, Rostock, Germany) for data acquisition with a boxcar width of 100 nm and an integration time of 2 s per scan. The percentage of light reflected was measured every 0.20 nm using an USB4000 miniature fiber optic spectrophotometer with a deuterium-tungsten halogen lamp to provide standardized illumination and a UV-visible reflection/backscatter probe with a wavelength range between 300 and 1100 nm (Ocean Insights, Rostock, Germany). The reflected light was collected at 45° to avoid specular reflectance. The probe was mounted on a prismatic holder and the distance to the corolla surface was 1 mm. A white standard (WS-1-SS White Standard, Ocean Optics Inc., Dunedin, FL, United States) was used to re-calibrate the equipment between each measurement in order to correct for possible shifts in spectrophotometer performance (Chittka and Kevan, 2005). Processing and visualization of the reflectance spectra were done using the pavo 2.2.0 package (Maia et al., 2019) of R software (R Core Team, 2020). Spectra were trimmed to the insect vision range, that includes human visible light and UV light (300–700 nm) using as.rspec function. Spectra were smoothed using local regression as implemented in the procspec function with span set to 0.25 because it was the minimum amount to remove spectral noise while preserving the original spectral shape. Measurements were averaged by individual and species, or by color type, individual and species for *Jaborosa laciniata* and *Jaborosa reflexa*, respectively, using the aggspec function.

### Corolla Reflectance Variation Among *Jaborosa* Species

We examined overall differences in reflectance spectra among the 11 *Jaborosa* species using Principal Component Analysis (PCA), which allowed us to summarize information of complex spectra in a few orthogonal variables while making no assumptions about the receiver’s visual system. We analyzed a total of 114 floral spectra that were centered to have a mean reflectance of zero and then binned into 10 intervals of 40-nm to obtain a dataframe suitable for PCA. Performing the PCA with standardized spectral data removes total reflectance as a dominant variable and allows comparing only the spectral shape (Cuthill et al., 1999; Maia et al., 2019).

### Evolution of Corolla Reflectance and Pollination Mode Among *Jaborosa* Species

We followed two different approaches to explore the evolution of corolla spectral shape among *Jaborosa* species. First, we represented overall variation in corolla color among species in a phylomorphospace (Chartier et al., 2014). Thus, a time-calibrated phylogeny enabled us to highlight trends in corolla color diversification in relation to species and pollination mode in the biplot resulting from the PCA analyses. The phylomorphospace was constructed using the mean loading values of the first two principal components (PCs) of each species and the MCC tree from the four-gene plastid dataset.

In a second step we estimated the ancestral reconstruction for corolla color and pollination mode. The best model of character evolution for the spectral shape (i.e., PC1 scores from the standardized PCA analysis) was determined using the *fitContinuous* function as implemented in “geiger” v.2.0.6 (Harmon et al., 2008). Three different models were fitted: Brownian motion (BM; Felsenstein, 1973), Ornstein–Uhlenbeck (OU; Hansen, 1997), and early burst (EB; Harmon et al., 2010). The BM model was chosen because, although it showed the same value as OU (σ^2^=40.93) it was identified as having the lowest AICc value. An estimation of the ancestral spectral shape was obtained using *anc.ML* function as implemented in “phytools” v.0.7-20 package (Revell, 2012). Model fitting of the pollination mode (discrete trait) was done using the *ace* function as implemented in “ape” v.5.3 (Paradis et al., 2004) under three different models: equal rates, symmetrical rates and all rates different. No significant differences were found after performing an ANOVA among the likelihood values of the different models. Thus, the equal rates model was chosen because it had the least number of estimated parameters. Character-state history among pollination modes was traced using a Bayesian stochastic character mapping approach using the *make.simmap* function from “phytools.” Multiple estimates were performed (*N* = 100) in order to provide the probability and confidence intervals of ancestral states at nodes (Bollback, 2006).

### Visual Modeling

Given that different animals process and perceive flower colors differently, we represented the corolla reflectance spectra in the visual perceptual spaces of the two main pollinator groups (nocturnal hawkmoths and saprophilous flies) and one occasional pollinator (the syrphid *Eristalis tenax*), covering the spectrum of pollinator functional groups recorded for the 11 *Jaborosa* species studied here(see section “Results”). This procedure allowed us to examine both how intra- and interspecific corolla color variation within the genus is perceived by these different functional groups (review by Renoult et al., 2017) and how such variation changed within the perceptual space of different pollinator functional groups during the diversification of the genus *Jaborosa*. We used two different kinds of models of color vision: the categorical perceptual spaces modeled for blowflies (*Lucilia* sp., Calliphoridae; Troje, 1993) and for hoverflies (*E. tenax*, Syrphidae; Lunau, 2014; Shrestha et al., 2016), respectively, and the Maxwell color triangle of a nocturnal hawkmoth (*Manduca sexta*, Sphingidae, Lepidoptera). We estimated quantum catches of each photoreceptor for the two model classes (fly and hawkmoth) incorporating a von Kries transformation that normalizes receptor quantum catches to the background (Maia et al., 2019). We used as background the average spectrum of the leaves from all *Jaborosa* species. According to the model proposed by Troje (1993), blowflies exhibit a categorical color vision system based on the relative excitations of the two pale-type (p+, p−) and two yellow type (y+, y−) receptors. The receptor of each pair that is stimulated more strongly determines which color the fly perceives. Thus, flies perceive four color categories: fly-UV (p+, y+), fly-blue (p−, y+), fly-yellow (p−, y−), and fly-purple (p+, y−). To plot corolla spectra from the *Jaborosa* species as loci in the fly vision models (blowfly and hoverfly), the quantum catches of each of the four photoreceptors were calculated by integrating the product of photoreceptor sensitivities, the standard D65 illumination and the corolla spectral reflectance. The photoreceptor sensitivities used for the blowfly *Lucilia* sp. follow Hardie and Kirschfeld (1983) and those for the hoverfly *E. tenax* follow Lunau (2014) and Shrestha et al. (2016). Stimuli with loci falling within the same color category are assumed to be indistinguishable to the fly (Troje, 1993; Arnold et al., 2009).

In contrast, hawkmoths have superposition eyes with three different photoreceptor types most sensitive in the ultraviolet (UV), blue (B), and green (G) ranges of the spectrum (White et al., 1994). We calculated the photoreceptor responses of *M. sexta* (UV = 357 nm, Blue = 450 nm, and Green = 520 nm; Bennett and Brown, 1985) and flower color loci were placed within the Maxwell color triangle. Each corner of the triangle corresponds to one of the three photoreceptor types of the moth eye and represents a pure color that only excites this receptor type. The location of a color locus in the triangle represents the relative excitation of the three receptor types by that floral sample (Balkenius et al., 2004). The light spectrum typical for sunset was used as ambient illumination (Henze et al., 2018), because hawkmoth foraging begins from sunset to dusk in this biome (Moré et al., 2006), although it can continue through the night (Vesprini and Galetto, 2000).

We estimated floral color conspicuousness for *M. sexta* under the Receptor Noise Limited (RNL) model of Vorobyev and Osorio (1998), in terms of chromatic and achromatic contrasts against the green leaves as background. This model has been behaviorally validated under different color stimulus choices for several species of diurnal and nocturnal hawkmoths (e.g., Kelber et al., 2002; Johnsen et al., 2006) including *M. sexta* (Kuenzinger et al., 2019). However, discrimination ability in the long wavelength range depends on both achromatic and chromatic differences (Telles et al., 2014, 2016). Chromatic contrast describes the color contrast that excludes luminance information while the achromatic contrast refers to the luminance difference between a flower color and its background. For chromatic contrast we used the following parameters: Weber fraction of 0.1 as empirically estimated for the tiger moth *Arctia plantaginis* (Henze et al., 2018); noise was set as “neural”; photoreceptor densities UV = 0.1, B = 0.23, L = 0.67 based on data from the ventral portion of the compound eye of *M. sexta* (White et al., 2003); and quantum catch was set to “Qi.” Achromatic contrast was calculated as the contrast produced in the long-wave photoreceptor (Henze et al., 2018). All calculations were performed using the pavo v.2.2.0 package (Maia et al., 2019) of R software (R Core Team, 2020). In order to determine whether samples of the *Jaborosa* species and pollination mode are discriminable in the hawkmoth’s color space, all pairwise chromatic distances measured in Just Noticeable Differences (JND) among species samples and pollination modes were calculated. A multi-response permutation procedure was then applied using a PERMANOVA with *bootcoldist* function where the observed pairwise mean color distance was compared with a distribution obtained by randomly assigning observed colors among samples. One thousand pseudo-values were obtained in this way, and the observed value was considered significant if it was greater than 95% of the pseudo-values. We considered this analysis to be adequate because it takes into account the multivariate nature of the data (Maia and White, 2018).

Finally, we estimated floral conspicuousness for the fly by calculating Euclidean distances among color loci as recently proposed by Hannah et al. (2019). Hannah et al. (2019) showed that color choices among different blue and yellow stimuli by the hoverfly *E. tenax* are mediated by a continuous monotonic function. These authors proposed that the Troje (1993) model of fly color processing could be a useful template for mapping how fly pollinators might discriminate flower colors from a background stimulus falling within the same quadrant.

### Evolution of Color Phenotype Across Environments

To test whether, along with evolutionary diversification in *Jaborosa*, the ability to colonize newly formed environments was concomitant with adjustment in flower color as is perceived by pollinators, we analyzed the association between transitions across environments and perceptual color axes. As formation of the new environments was synchronous with the uplifting of the Central Andes and rain shadowing of its eastern slopes during the last 10 Ma (see section “Discussion”), the initial humid lowland forests and grasslands were succeeded by dry mid-altitude scrub and subsequently by cold alpine and high latitude cold deserts (Salzmann et al., 2008; Strelin et al., 2017). For the purpose of analysis, we classified the area now occupied by *Jaborosa* in three main environmental zones that are consistent with groups of widely recognized ecoregions in South America (see Olson et al., 2001 and Supplementary Table 2): (1) humid lowland zone, (2) dry lowland and foothill zone, and (3) alpine and high latitude zone. These zones range in pollinator environment from hawkmoth-dominated to fly-dominated pollinator climates (see Arroyo et al., 1982; Elberling and Olesen, 1999; Devoto et al., 2005; Chacoff et al., 2012; Sazatornil et al., 2016; Moré et al., 2019). This classification is constrained by the relevant groups of pollinators for *Jaborosa* species and does not include Hymenoptera and vertebrates (bats, hummingbirds, and rodents) that are also important pollinators in the dry lowland and high altitude habitats of southern South America, respectively (Strelin et al., 2017; Dellinger et al., 2019).

The ability of extant *Jaborosa* species and their reconstructed ancestors to occupy different environmental zones was explored in a threshold model which uses Bayesian Markov chain Monte Carlo (MCMC) to sample the liabilities from joint posterior probability distributions (Wright, 1934; Revell, 2014). The threshold model is inherently ordered, thus the three environmental zones were ordered according to their chronological appearance as (1) < (2) < (3). To test whether evolutionary changes in environmental occupancy were associated with orderly transitions along pollinator’s perceptual axes, we carried out phylogenetic generalized least squares (PGLS) correlations between liabilities to occupy environments and perceptual color vectors. We constructed color vectors using as variables the relativized photon catches (Qi) for hawkmoth and the Euclidean distances for flies, which were obtained from the above visual models. We undertook a Principal Component Analyses (PCA) of Qi values (hawkmoth) and Euclidean distances (blowfly) and the scores of the PC1 axis were used as the color vector. Two evolutionary models were fitted for correlated evolution: BM (Felsenstein, 1973) and OU (Hansen, 1997). The OU model was chosen because it was identified by AIC as the best fit. Analyses were carried out with functions *ancThresh* and *phyl.vcv* in phytools 0.7-20 package (Revell, 2012) of R software (R Core Team, 2020).

## Results

### Pollinators

The *Jaborosa* species studied were pollinated by insects that could be assigned to three functional groups: saprophilous flies (F), nocturnal hawkmoths (H), and generalized pollination (G). We recorded mostly saprophilous flies as pollinators of five *Jaborosa* species: *J. laciniata*, *Jaborosa leucotricha*, *Jaborosa magellanica*, *Jaborosa rotacea* and *Jaborosa sativa.* Flies belonged to three superfamilies: Muscoidea (Anthomyiidae and Muscidae), Oestroidea (Calliphoridae, Sarcophagidae, and Tachinidae) and Tabanoidea (Tabanidae) and consistently acquired pollen on the dorsal surfaces of their thoraces (nototribic deposition). *J. integrifolia* was pollinated at night by *Manduca tucumana* (Lepidoptera: Sphingidae). A total of 323 nocturnal hawkmoths belonging to 14 species were captured at two western populations of *J. integrifolia*; El Fuerte and Termas de Reyes. Of this sample, seven individuals of the most abundant hawkmoth species, *M. tucumana* (*N* = 125, mean proboscis length = 79.42 mm), carried pollen loads of *J. integrifolia* on their probosces. We did not record any hawkmoth visits nor the presence of moth scales on floral stigmas in eastern populations (Diamante and Victoria) of *J. integrifolia* during two different years. However, a previous study conducted near our field sites documented rare but effective hawkmoth pollination of *J. integrifolia* (Vesprini and Galetto, 2000). During daytime, a melyrid beetle (*Astylus quadrilineatus*) was commonly observed visiting flowers of both *J. integrifolia* and *Jaborosa runcinata* in two populations (Diamante and Victoria) where these species grow in sympatry. Beetles were observed in high numbers within the flowers (up to 10 individuals were recorded in a single flower of *J. integrifolia*) either feeding on pollen or copulating. The presence of moth scales on the flower stigmas of *Jaborosa odonelliana* and *J. runcinata* provides indirect evidence of moth visitation at night. *J. reflexa* demonstrated generalized pollination by small insects, including syrphid and sarcophagid flies and halictid bees. Ants were observed patrolling the leaves and flowers of two species (*Jaborosa bergii* and *J. rotacea*) but did not contact the anthers and stigmatic surfaces. Finally, we did not observe any floral visitors for two of the studied species, *J. bergii* and *J. odonelliana*, neither did we observe any visitors for the green (polymorphic) *J. laciniata* flowers nor the maroon flowers of *J. reflexa* (Supplementary Table 1).

### Corolla Reflectance Variation

**FIGURE 2:**
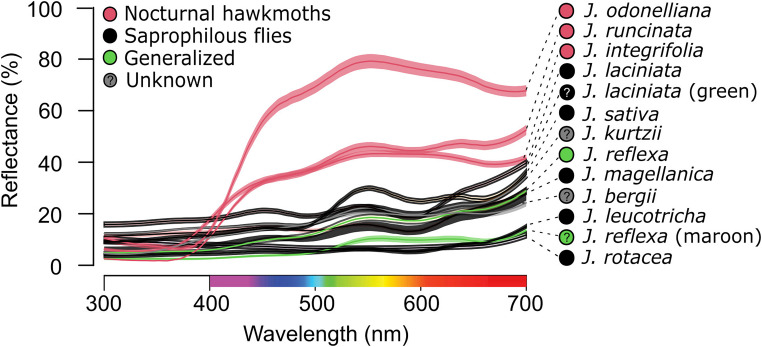
Averaged corolla reflectance of *Jaborosa* species. Lines and shadowed areas show average spectra and standard deviation per species. Colors show pollination mode: pink for hawkmoths, black for saprophilous flies, green for generalized, and gray for unknown pollinators, respectively. Question marks in *J. laciniata* green morph and *J. reflexa* maroon morph stand for unknown pollinators.

We recorded variation in corolla reflectance spectra both among *Jaborosa* species and among individuals within species (Figure 2). Species pollinated or putatively pollinated by nocturnal hawkmoths (*J. integrifolia*, *J. odonelliana*, and *J. runcinata*) showed corollas with maximum reflectance (50–80% of white standard) between 450 and 700 nm, absorbing in the UV-region of the spectrum, between 300 and 399 nm, and perceived as white by human vision. Diurnal species showed corollas with very low overall reflectance and maximum reflectance of approximately 20% of the white standard. Saprophilous fly-pollinated species were maroon to black in color as perceived by human vision (*J. laciniata*, *J. leucotricha*, *J. rotacea*, *J. magellanica*) or pale green freckled with dark blotches (*J. sativa*). Two species showed intraspecific variation in corolla color. *J. laciniata* was polymorphic, with flower colors varying among individuals between black or more rarely green. *J. reflexa* was heteromorphic in flower coloration, with individuals showing flowers of the same age either yellowish to pale green or maroon (Figures 1, 2). Thus, in these two species, we considered flower types separately for subsequent analyses.

**FIGURE 3:**
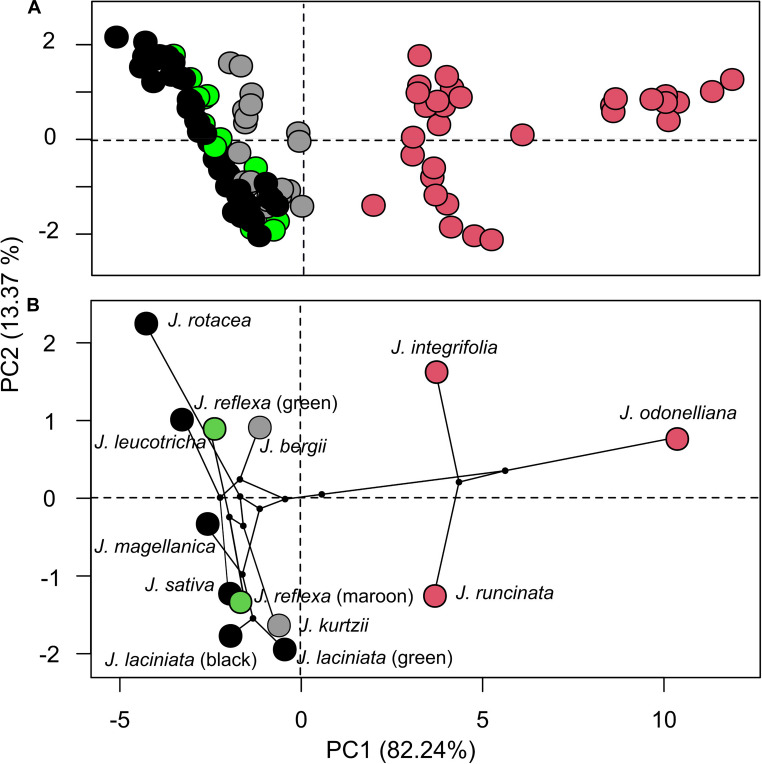
Overall variation in corolla reflectance among *Jaborosa* species. **(A)** Spectral shape scatter plot from a PCA analysis, each point represents an individual. **(B)** Projection of the *Jaborosa* phylogenetic tree into the color space defined by the first two axes, each point represents the species mean. Different colors show pollination mode: pink for hawkmoths, black for saprophilous flies, green for generalized, and gray for unknown pollinators, respectively. Question marks in *J. laciniata* green morph and *J. reflexa* maroon morph stand for unknown pollinators.

The first two PCs of the PCA accounted for 95.61% of total variation in spectral shape among *Jaborosa* species and among individuals within each species. Variation in flower reflectance was detected in PC1 according to pollination mode, loadings of the three species with nocturnal hawkmoth-pollinated flowers clustered together and separated from the plant species with diurnal flowers (five saprophilous fly-pollinated species, the one with generalized pollination and the two species with unknown pollinators; Figure 3A). The phylomorphospace showed that variation in reflectance spectra is associated with clades, with the three species comprising the humid lowland clade showing high values of PC1 loading scores and the Andean clade with low values of PC1 loading scores (Figure 3B).

### Evolution of Corolla Reflectance and Pollination Mode Among *Jaborosa* Species

Our results show that diversification within the Andean clade is associated with a pollinator shift from nocturnal hawkmoths to saprophilous flies and occurred in concert with changes in corolla coloration – from white flowers to either maroon and black flowers or pale green freckled with dark blotches – during the initial diversification of *Jaborosa* ancestors across southern South America (Figure 3B).

**FIGURE 4:**
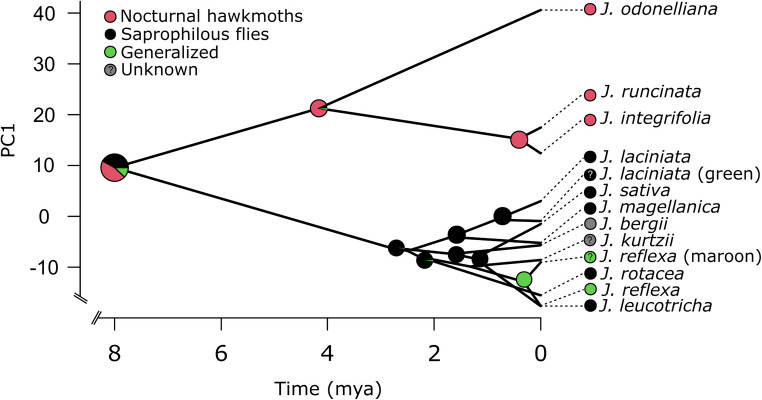
Reconstruction of ancestral corolla color (PC1 loading scores from a standardized PCA analysis) and pollination mode in the genus *Jaborosa*. The phylogenetic tree was projected in the space defined by corolla spectral shape (*y*-axis) and divergence time (*x*-axis). Pie charts on each node show the posterior probability of each pollination mode retrieved by 100 stochastic character mappings. Question marks in *J. laciniata* green morph and *J. reflexa* maroon morph stand for unknown pollinators.

The most recent common ancestor (MRCA) of extant *Jaborosa* was reconstructed as bearing corollas with low reflectance in the medium- and long-wavelength regions and absorbing in the UV and short-regions (perceived as whitish to pale greenish by humans). The ancestor of the lowland clade was reconstructed as bearing corollas with high reflectance in the overall short-, medium-, and long-wavelength ranges (from 400 up to 700 nm) and absorbing in the UV region (perceived as white by humans) while the ancestor of the Andean clade was reconstructed as bearing corollas with low overall reflectance (perceived as maroon by humans). Reconstruction of pollination mode in the MRCA of extant *Jaborosa* was ambiguous. The MRCA of the lowland clade was reconstructed as being mostly pollinated by hawkmoths about 4.2 Ma while the MRCA of the Andean clade was reconstructed as being pollinated by saprophilous flies about 2.8 Ma. Thus, pollination mode (hawkmoths vs. saprophilous flies) is contingent with changes in the corolla spectral shape. Finally, generalized pollination in *J. reflexa* appears to have evolved from a specialized saprophilous fly-pollinated ancestor (Figure 4).

### Floral Color Discrimination in Pollinator Visual Spaces

**FIGURE 5:**
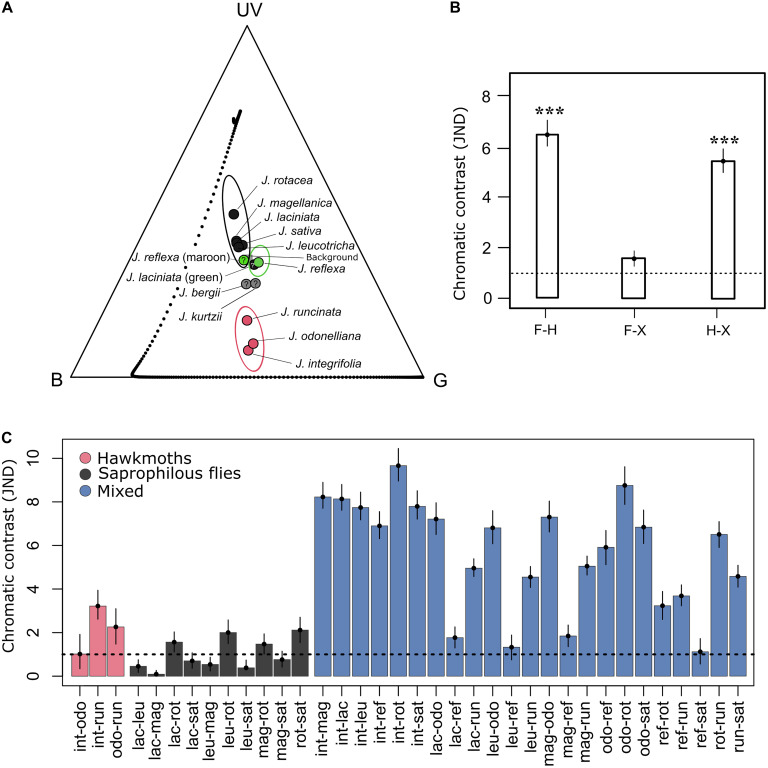
Color loci of *Jaborosa* flowers in the trichromatic model of *Manduca sexta* and the bootstrapped, noise-corrected chromatic distances among *Jaborosa* species and pollination modes. **(A)** The vertices of the Maxwell triangle represent colors that excite only one of the three receptor types (UV, B and G). **(B)** Bars show mean chromatic distances among pollination modes. Whiskers show 95% confidence intervals for color discrimination (F, flies; H, hawkmoths; X, generalized pollination by small insects). The dashed line represents the theoretical discrimination threshold of one JND. ***Show a significant distance at *P* < 0.001 (PERMANOVA test). **(C)** Bars show mean chromatic distances between pairs of *Jaborosa* species ordered according to their pollination mode (pink for hawkmoth pollinated, gray for saprophilous fly-pollinated, blue for hawkmoth- vs. saprophilous fly-pollinated, hawkmoth vs. generalist and saprophilous fly-pollinated vs. generalist).

Floral color loci in the *M. sexta* visual space for the three hawkmoth-pollinated species were clustered together in the region between blue and green photoreceptors and distant from the central zone, such that they would be perceived as colorful objects against the leaf background in the color space of *M. sexta* (Figure 5A and Supplementary Figure 1). The remaining *Jaborosa* species clustered around the central zone, such that they would be perceived by hawkmoths as colors very similar to the leaf background.

We detected a significant discrimination in corolla color between pollination modes in the visual space of *M. sexta.* The highest chromatic contrast was observed between hawkmoth- and saprophilous fly-pollinated species and between hawkmoth-pollinated species and the generalist-pollinated *J. reflexa*. No differences in chromatic contrast were detected among species pollinated by saprophilous flies and the generalist species (Figure 5B). When considering pairwise discrimination between *Jaborosa* species, chromatic distances were small and, in general, not significantly different within species sharing the same pollination mode but were significantly larger among species differing in pollination mode (Figure 5C). Thus, our results suggest a conservatism or convergence in corolla coloration within pollination modes. With regard to achromatic conspicuousness for hawkmoths, all achromatic contrasts among pollination modes were higher than 0.1 and thus were easily discriminable (Supplementary Figure 2).

**FIGURE 6:**
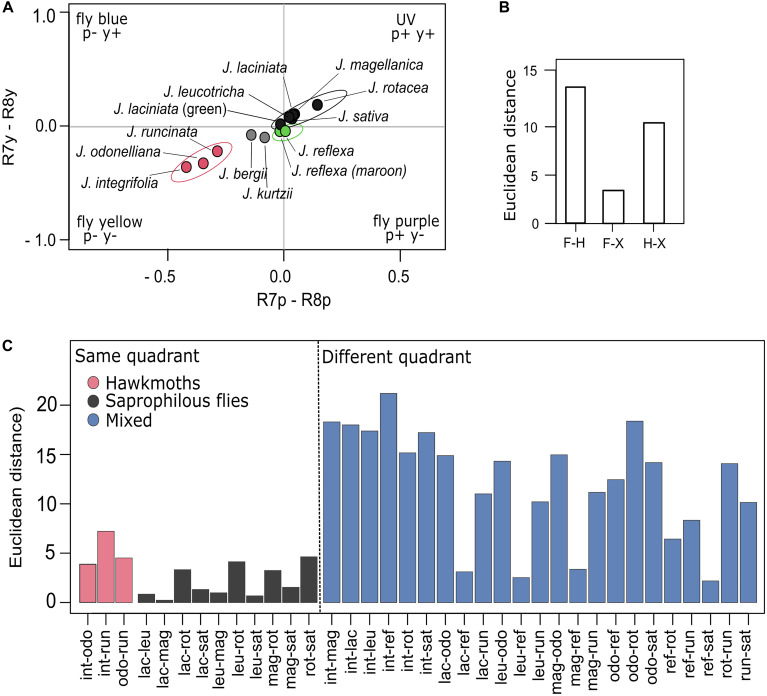
Colors of *Jaborosa* flowers according to how they would be perceived by a blowfly. **(A)** COC for *Lucilia* sp. Colors in the same quadrant are not discriminated as different by the fly. **(B)** Bars show mean color distances (Euclidean distances) among pollination modes (F: Flies, H: Hawkmoths, X: Generalized pollination by small insects). **(C)** Bars show mean color distances (Euclidean distances) between pairs of *Jaborosa* species with known pollinators. Species are ordered according to their pollination mode (pink for hawkmoth pollinated, gray for saprophilous fly-pollinated, and blue for hawkmoth- vs. saprophilous fly-pollinated, hawkmoth vs. generalist and saprophilous fly-pollinated vs. generalist). The dotted vertical line separates those *Jaborosa* species pairs that are in the same quadrant (left) from the ones that are in different quadrants (right).

Floral color loci for the saprophilous fly-pollinated *Jaborosa* species all fall within the fly-UV quadrant of the visual spaces for the blowfly *Lucilia* sp. and the hoverfly *E. tenax* (Figure 6A and Supplementary Figure 1). Thus, neither carrion-flies nor hoverflies would be able to distinguish among *Jaborosa* species located in the same quadrant. In contrast, hawkmoth-pollinated species together with the green morph of *J. reflexa* – pollinated by a mixed array of pollinators-, *J. bergii* and the green morph of *J. laciniata* are placed within the fly-green quadrant (Figure 6A and Supplementary Figure 1).

We detected a significant discrimination in corolla color between pollination modes in the visual space of *Lucilia* sp. The highest Euclidean distance was observed between saprophilous fly- and hawkmoth-pollinated species, followed by the distances between the generalist-pollinated *J. reflexa* and hawkmoth-pollinated or saprophilous fly-pollinated *Jaborosa* species (Figure 6B). When considering pairwise discrimination between *Jaborosa* species, Euclidean distances were small among species sharing pollination mode (i.e., hawkmoths or saprophilous flies) but were significantly larger among species differing in pollination mode, given that most of them fell within different quadrants of blowfly visual space (Figure 6C).

### Evolution of Color Phenotype Across Environments

**FIGURE 7:**
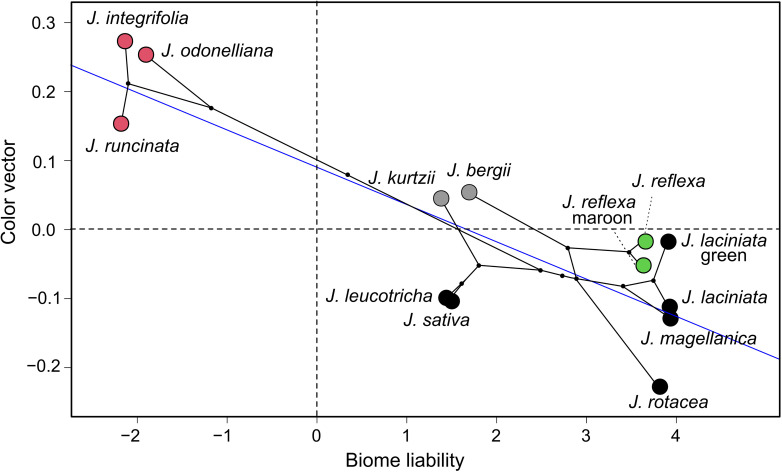
Evolutionary transitions along the color vision axis. Phylogenetic generalized least squares correlation (PGLS) between environment occupancy (represented as liabilities, *x*-axis) and perceptual color vector of the nocturnal hawkmoth *Manduca sexta*. Blue line represents the slope. Different colors show pollination mode: pink for hawkmoths, black for saprophilous flies, green for multiple, and gray for unknown pollinators, respectively. See text for further explanations.

Evolutionary transitions along the color vision axes were significantly associated with the ordered transitions in environmental occupancy across the phylogeny of the studied *Jaborosa* species. This was true both for hawkmoth and fly visual model vectors (Supplementary Table 3, Figure 7, and Supplementary Figure 3).

## Discussion

Our results revealed a striking, non-random pattern of flower coloration associated with two primary modes of pollination in the genus *Jaborosa*: unrewarding (brood-site deceptive) flowers with inconspicuous and low reflective corollas mainly pollinated by saprophilous flies vs. nectar-rich flowers with conspicuous and highly reflective corollas pollinated by nocturnal hawkmoths. These utterly different pollination modes are separated geographically, as saprophilous fly pollination is associated with species growing in high altitude (Andes) or latitude (Patagonian steppe) environments, whereas nocturnal pollination by hawkmoths is associated with species growing in humid subtropical lowlands. Species with intermediate coloration patterns showed generalist pollination by small insects including saprophilous flies or unknown pollinators and are found in intermediate geographic locations such as dry lowland and foothill zones (Figure 1).

### Flower Conspicuousness and Pollination Mode in *Jaborosa*

Similar patterns of corolla reflectance to those found here for sphingophilous *Jaborosa* species (i.e., absorbing in the UV region and being highly reflective between 400 and 700 nm) were reported for other hawkmoth-pollinated plant species across the Americas, where nocturnal hawkmoths are widely attracted to flowers that appear “white” to humans (Haber and Frankie, 1989; White et al., 1994; Raguso et al., 2003). Nocturnal hawkmoths are major pollinators in South America where they visit hundreds of flowers each night to fuel their high energetic demands by drinking nectar (Sazatornil et al., 2016). Some nocturnal hawkmoths can discriminate flower coloration at starlight intensities at which humans and honeybees are colorblind (Kelber et al., 2002) but they rely on both olfactory and visual cues to recognize, approach and feed from flowers (Raguso and Willis, 2005; Goyret et al., 2007). *M. sexta* also can utilize achromatic (corolla brightness) signals, above-ambient CO_2_, humidity and mechanosensory cues (corolla morphology) to successfully drink nectar from flowers (Goyret and Raguso, 2006; Goyret, 2010; Goyret and Kelber, 2012; review by Stöckl and Kelber, 2019). It is known that innate feeding behavior is mediated by blue photoreceptors in the nocturnal hawkmoth *M. sexta* (Cutler et al., 1995), which is able to switch color preferences under different photic conditions (Kuenzinger et al., 2019) or after training experience (Goyret et al., 2008) suggesting that both sensory plasticity and color learning capabilities play important roles in flower-foraging behavior.

Similar patterns of corolla reflectance to those found here for saprophilous fly-pollinated *Jaborosa* species have been reported for other sapromyiophilous plant species across the world (Urru et al., 2011; Chen et al., 2015; Du Plessis et al., 2018). Forensic studies indicate that the coloration of dead animal bodies is dynamic as their carcasses decay. Dull red *livor mortis* is observed after several hours following death, when greenish discoloration becomes brownish or black (Chen et al., 2015). We measured the full range of these color changes in the corollas of *Jaborosa* species pollinated by saprophilous flies (Figure 1). Saprophilous flies use both olfactory and visual cues to find oviposition substrates (Wall and Fisher, 2001; Chen et al., 2015). We previously demonstrated that floral scent, specifically sulfur-containing volatile compounds, plays an important role as an attractant of saprophilous flies to flowers of *J. rotacea* and *J. laciniata* in natural settings (Moré et al., 2013, 2019).

Evolutionary studies often focus on the evolution of specialized pollination systems while very interesting patterns may emerge from the evolution of generalized pollination from specialized pollination (Waser et al., 1996; Johnson and Steiner, 2000). Interestingly, we found that the evolution of generalized pollination mode in *J. reflexa* is likely to have evolved from a specialist ancestor pollinated by saprophilous flies (Figure 4). One possible mechanism to explain the evolution of generalist pollination in *J. reflexa* is a geographic range extension since is the only *Jaborosa* species growing in some areas of the vast Patagonian steppe. This species showed an interesting pattern of intra-specific variation in flower phenotype that merits further study.

Floral color conspicuousness (in terms of chromatic and achromatic contrast against the background; Schmidt et al., 2004) may influence pollinators’ detection of *Jaborosa* flowers either for nectar foraging (hawkmoths, small insect) or oviposition deceit (saprophilous flies). Difference in color conspicuousness was high and significant between the sphingophilous *Jaborosa* species and the diurnal pollinated species (as expected for *M. sexta*; Figure 5B and Supplementary Figure 3), but was small and not significant between the diurnal pollinated species (saprophilous fly-pollinated vs. generalist by small insects), as expected for *Lucilia* sp. flies (Figure 6B). Significantly greater conspicuousness (chromatic and achromatic) in the hawkmoth-pollinated species ensures that flowers could be detected by hawkmoths even under starlight conditions.

When taking into account color conspicuousness differences between species pairs, it is interesting that within the sphingophilous clade the short-tubed *J. runcinata* is perceived as different from the other two long-tubed species – *J. integrifolia* and *J. odonelliana* – in the visual space of *M. sexta* (JND > 1; Figure 1). It is possible that character displacement in tube length and corolla coloration represent prezygotic barriers to pollen flow between these taxa. This is relevant because *J. runcinata* and *J. integrifolia* are sympatric at our lowland study sites, where plants with morphologically intermediate flowers can be found (unpublished data). A similar trend was observed in the nightshade genus *Iochroma* where taxa that occur in sympatry occupy a significantly larger volume of color space than those in allopatry, suggesting that competition among close relatives may commonly underlie floral divergence, especially in species-rich habitats where congeners frequently co-occur (Muchhala et al., 2014).

On the other hand, the smallest color conspicuousness differences were observed among saprophilous fly-pollinated species. These species, which cluster in the UV quadrant of the *Lucilia* sp. color space, would be indistinguishable by the flies, given the visual model used. Interestingly, this is the quadrant in which flies perceive the skin color of dead cattle in a stage of *livor mortis* (Chen et al., 2015), suggesting that convergence in flower color is the result of mimicry. It was revealed that variation in coloration between different parts of the inflorescence of *Amorphophallus konjac* is associated with differential attraction of saprophilic flies (Chen et al., 2015). This niche partitioning between groups of calyptrate flies (e.g., sarcophagids vs. calliphorids) was also reported in the orchid *Satyrium pumilum*, in this case by flower size and scent intensity rather than coloration pattern of the corolla (Van Der Niet et al., 2011). We suspect that similar niche partitioning is occurring in *Jaborosa*, given observed variation in flower coloration, size, morphology, and scent composition and intensity and the diversity of fly visitors (Supplementary Table 1).

### Geological Events Underlying Pollination Climates in *Jaborosa*

Andean orogeny and the ecological changes that followed have promoted diversification in plant and animal lineages since the Early Miocene (Luebert and Weigend, 2014). Studies on the paleobotany and geology of South America suggest that up to half of the Central Andes uplift (i.e., a rise from 2000 m to the present 4000 m) has taken place during the last 10 Ma. Divergence of the MRCA of *Jaborosa* in the two main clades occurred approximately 8 mya ago during the late Miocene, when geological evidence suggests that the Andes were still relatively low (400–2500 m) and climate was warm and humid at southern latitudes (Figure 4). The split of these two major clades has strong geographical structure, suggesting early isolation of these two ancestral lineages (Figure 1).

Interestingly, there is paleo-environmental evidence supporting the existence of a geographic barrier at the time of early divergence in *Jaborosa*. Studies have shown that during the middle and late Miocene (ca. 10–17 mya) three successive Atlantic marine transgressions, informally known as the “Paranean Sea,” resulted in a flooded area in southern South America possibly separating the ancestors of these lineages (Tambussi and Degrange, 2012; see Svensson et al., 2016 for a similar example in North America). At the same time, cold desert climate covered the plains east of the Southern Andes and gave way during the Late Miocene (*ca.* 8 Ma) to the Patagonian steppe, which in the present features near-alpine climatic conditions (see Barreda et al., 2008). Concomitant with Andean orogeny, glaciation began in the late Pliocene (*ca.* 3 Ma), placing the origins of alpine Andean vegetation at ca. 3–5 Ma, making it the youngest natural ecosystem of South America (Graham, 2009). During the Pliocene, the Puna-Altiplano Plateau rose from 2500 to 4000 m.a.s.l. (∼5 Ma to present; Barnes and Ehlers, 2009) and small dust particles were transported by dust storms from the Puna-Altiplano Plateau to the Pampas grasslands and deposited by rain (∼3 Ma to present; Gaiero et al., 2013).

### Pollinators’ Perceptual Context of Flower Color Diversification

Extensive evidence has demonstrated that the latitudinal and altitudinal distribution of floral syndromes is influenced by the distributions of their respective pollinator guilds (Ollerton et al., 2006). In this context, the Andes provide a unique geographic scenario in southern South America, under which several pollinator transitions have been recognized (Pérez et al., 2006; Smith et al., 2008; Schlumpberger et al., 2009; Strelin et al., 2017; Dellinger et al., 2019). Our findings suggest that floral diversification in the genus *Jaborosa* could have occurred in concert with a geographic shift from Subtropical lowlands (Chaco and Pampas grasslands) to cool-temperate regions with marked changes in daily temperatures (Monte and Prepuna deserts, high mountain Andes and Patagonian steppe) accompanied by geographical differences in pollinator availability, specifically reduced abundance of hawkmoths. The harsh environmental conditions in high elevation Andean habitats (low temperatures, short growing seasons, and strong winds) generally reduce the spectrum of possibilities for biotic pollination. Colonization of these new habitats with colder temperatures reduce visitation by long-tongued hawkmoths because they are endothermic insects that rarely fly above 1300 m in Subtropical Argentina (Moré et al., 2014). Hawkmoth diversity is higher in subtropical lowland areas where ambient temperatures at dusk are moderate, and where nocturnal hawkmoths constitute a substantial component of the pollinator fauna (Cruden et al., 1976; Haber and Frankie, 1989; Sazatornil et al., 2016). Out of 117 hawkmoth species recorded from Argentina, only the short-tongued *Hyles euphorbiarum* (proboscis length shorter than 30 mm), is consistently recorded beyond 40° south latitude (Moré et al., 2014). Interestingly, other plant lineages with significant amounts of sphingophily, such as *Nicotiana* (Solanaceae) and the family Cactaceae, show the same geographic and altitudinal patterns described here (Goodspeed, 1954; Schlumpberger and Renner, 2012). It is worth mentioning that in addition to metabolic limits, other factors may be involved in the distribution of hawkmoth fauna. In South Africa, the long-tongued *Agrius convolvuli* and its associated plant guild are only distributed in the eastern subtropical regions and are absent from the western Cape region and the arid south-west (Johnson and Raguso, 2016). This appears to be a general pattern because the Cape fynbos vegetation lacks families such as Rubiaceae, Balsaminaceae, Vitaceae, Convolvulaceae, and Loganiaceae that comprise the larval food plants for many hawkmoths (Attie et al., 2010).

Interestingly, the converse situation is not observed, given that the geographic distribution of saprophilous flies is not limited by ambient temperature and metabolic energetics. Calyptrate flies can tolerate high temperatures and are broadly distributed across geographical domains, to the extent that some species (e.g., *Lucilia sericata*) are used as nearly universal indicators in forensic studies (Mulieri et al., 2010). This asymmetry in pollinator distribution, which defines pollinator climate for *Jaborosa*, is highlighted by a simple experiment in which volatile sulfides typical of saprophilous fly pollinated *J. laciniata* were added to the large, white flowers of *J. integrifolia* in its lowland habitat during daytime. Within minutes of scent augmentation, calliphorid flies approached, landed upon, and attempted to feed from the sphingophilous flowers of *J. integrifolia* (Moré et al., 2019). Thus, the putative direction of evolution in Figure 4 (e.g., from a sphingophilous ancestry) suggests a transition to fly pollination in colder, drier habitats devoid of hawkmoths, whereas saprophilous flies are present in all habitats occupied by *Jaborosa* species.

Pollination by saprophilous flies becomes substantial in the high-mountain Andes where pollinator limitation strongly limits the success of self-incompatible plant species (Arroyo et al., 1982; Pérez et al., 2009). Particularly, the flesh-fly genus *Microcerella*, recorded here as pollinators of the Patagonian species *J. magellanica* and *J. reflexa*, show greater diversity in arid and high-altitude environments of South America (Mulieri and Mariluis, 2009). Saprophilous fly-pollinated species of *Jaborosa* are restricted to regions where species pollinated by long-tongued hawkmoths are absent (neither beyond 40° South latitude nor above 3000 m in the Andes. Figure 1). Although brood-site deceptive fly-pollinated species of *Jaborosa* are restricted to the such cold-temperate environments, other plant lineages pollinated or putatively pollinated by saprophilous flies are distributed in warm-temperate and subtropical areas of South America, such as *Aristolochia* spp. (Aristolochiaceae), *Synandrospadix vermitoxicus* (Araceae), *Pleurothallis* spp. (Orchidaceae) and *Gonolobus* spp. (Apocynaceae), and plants with similar reproductive strategies are found in warm/subtropical biomes worldwide (review by Jürgens et al., 2006; Urru et al., 2011; Jürgens et al., 2013).

## Conclusion

Our results revealed two major evolutionary themes for the flower color diversification pattern in the South American genus *Jaborosa*. The first is a “warm subtropical sphingophilous clade” composed of three hawkmoth-pollinated species found in humid lowland habitats, with large white flowers that clustered together in the “blue-green” region and distant from the central zone in the visual space of the model hawkmoth *M. sexta* visual space. Thus, they are perceived as colorful objects against the vegetative background in hawkmoth color space (Figure 5). The second is a “cool-temperate brood-deceptive clade” composed of largely fly-pollinated species found at high altitudes (Andes) or latitudes (Patagonian Steppe), with small, dark flowers that clustered together in the UV quadrant in the visual space of the model blowfly *Lucilia* sp. (Figure 6). Our findings, based on multivariate analyses of reflectance spectra, ancestral reconstruction of flower color and pollination mode and comparative methods (Figures 3, 4, 7) suggest that the ability of plants to colonize newly formed environments during Andean orogeny and the ecological changes that followed were concomitant with adjustment in flower color as perceived by different pollinator groups. Adaptation to habitat and pollination mode are inextricably linked in the history of this South American plant lineage.

## Data Availability Statement

The datasets presented in this study can be found in online repositories. The names of the repository/repositories and accession number(s) can be found below: The flower reflectance spectra analyzed in this study will be available at the Floral Reflectance Database (ID #4383–#4505 at http://www.reflectance.co.uk/).

## Author Contributions

MM, AAC, and RAR contributed to the design and implementation of the research. MM, ACI, MED, and AAC contributed to the analysis of the results. All authors contributed to the writing of the manuscript.

## Conflict of Interest

The authors declare that the research was conducted in the absence of any commercial or financial relationships that could be construed as a potential conflict of interest.

## Supplementary Material

Supplementary Figure 1 Color loci of all measured *Jaborosa* individuals in the trichromatic model of the nocturnal hawkmoth *Manduca sexta* (upper panel) and how they would be perceived by the blowfly *Lucilia* sp. (bottom left) and the hoverfly *Eristalis tenax* (bottom right).

Supplementary Figure 2 Mean differences in color conspicuousness (achromatic contrast) among pollination modes (F, flies; H, hawkmoths; X, generalized pollination by small insects). The dashed line represents the theoretical discrimination threshold of 0.1 JND.

Supplementary Figure 3 Evolutionary transitions along the color vision axis. Phylogenetic generalized least squares correlation (PGLS) between environment occupancy and perceptual color vector of the blowfly *Lucilia* sp. (see text for further explanations). The dashed horizontal line (at −0.9 in the *y*-axis) corresponds to the center of the fly color space where all the quadrants coincide. Thus, *Jaborosa* species that are in the p−y− quadrant are shown above the line, and those *Jaborosa* species that are in the p+y+ quadrant are shown below the line.

Supplementary Table 1 *Jaborosa* species studied for the corolla reflectance analyses and visual models. We provide information about pollinator observations (direct observation, ligh trapping and hawkmoths’ scales analysis) in different localities. Asterisks show confirmed pollinators.

Supplementary Table 2 Environmental zones classification used in the PGLS analyses: humid lowland zone (1), dry lowland and foothill zone (2), and alpine and high latitude zone (3).

Supplementary Table 3 Phylogenetic generalized least squares model among *Jaborosa* species between environmental occupancy and flower color expressed in hawkmoth and blowfly vision models.
