# Supplementary figures and images for: Flower Diversification Across “Pollinator Climates”: Sensory Aspects of Corolla Color Evolution in the Florally Diverse South American Genus Jaborosa (Solanaceae)
Source: Front Plant Sci. 2020 Dec 7;11:601975. doi: 10.3389/fpls.2020.601975 (PMC7750315; doi:10.3389/fpls.2020.601975)

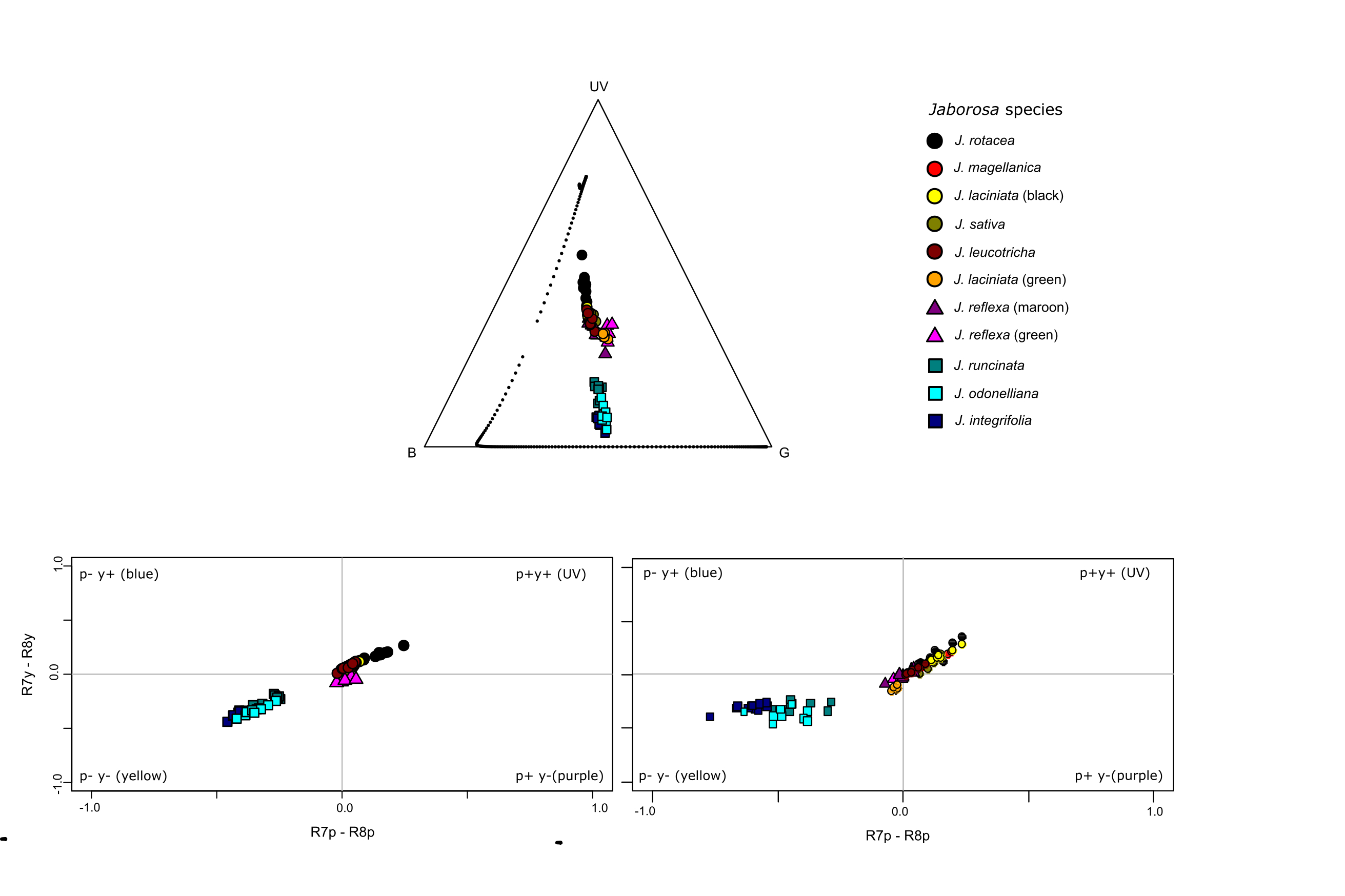

Supplement: Supplementary Figure 1 — Color loci of all measured Jaborosa individuals in the trichromatic model of the nocturnal hawkmoth Manduca sexta (upper panel) and how they would be perceived by the blowfly Lucilia sp. (bottom left) and the hoverfly Eristalis tenax (bottom right). [file Image_1.JPEG]

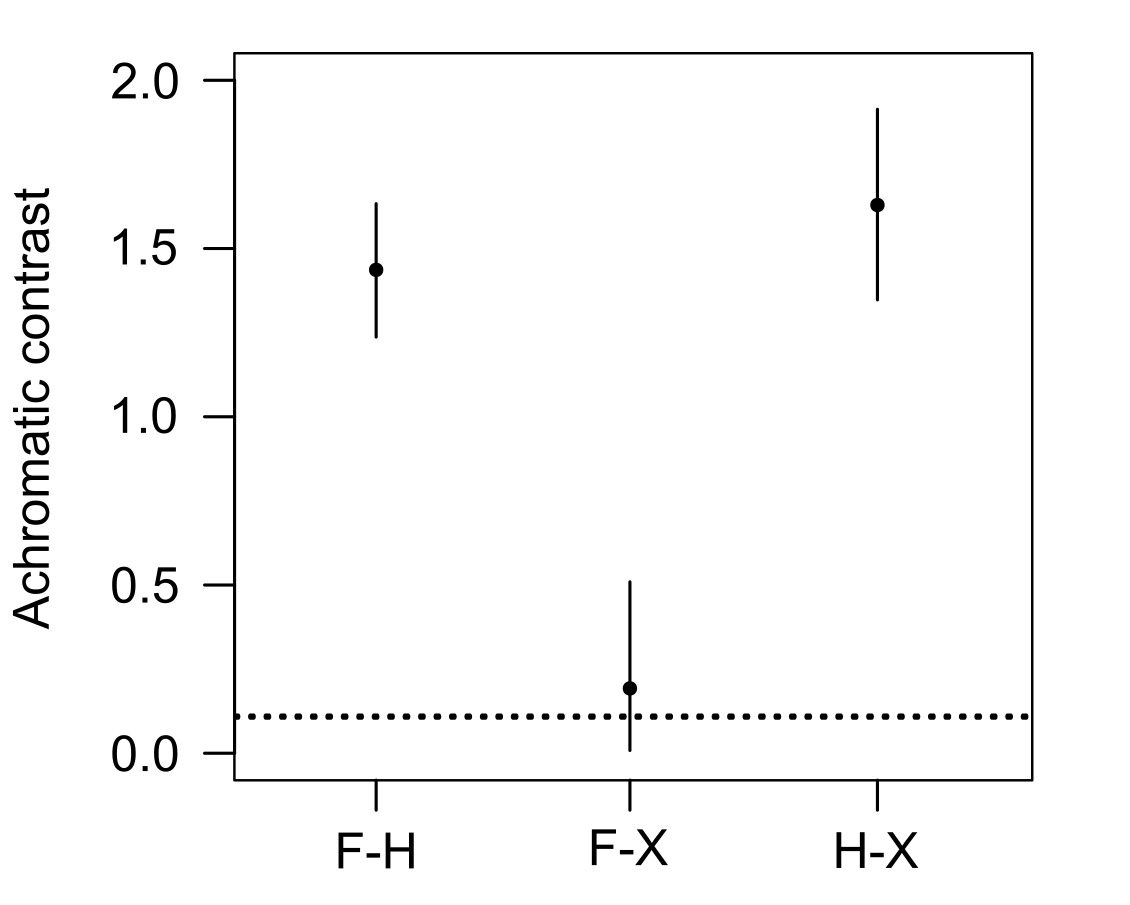

Supplement: Supplementary Figure 2 — Mean differences in color conspicuousness (achromatic contrast) among pollination modes (F, flies; H, hawkmoths; X, generalized pollination by small insects). The dashed line represents the theoretical discrimination threshold of 0.1 JND. [file Image_2.JPEG]

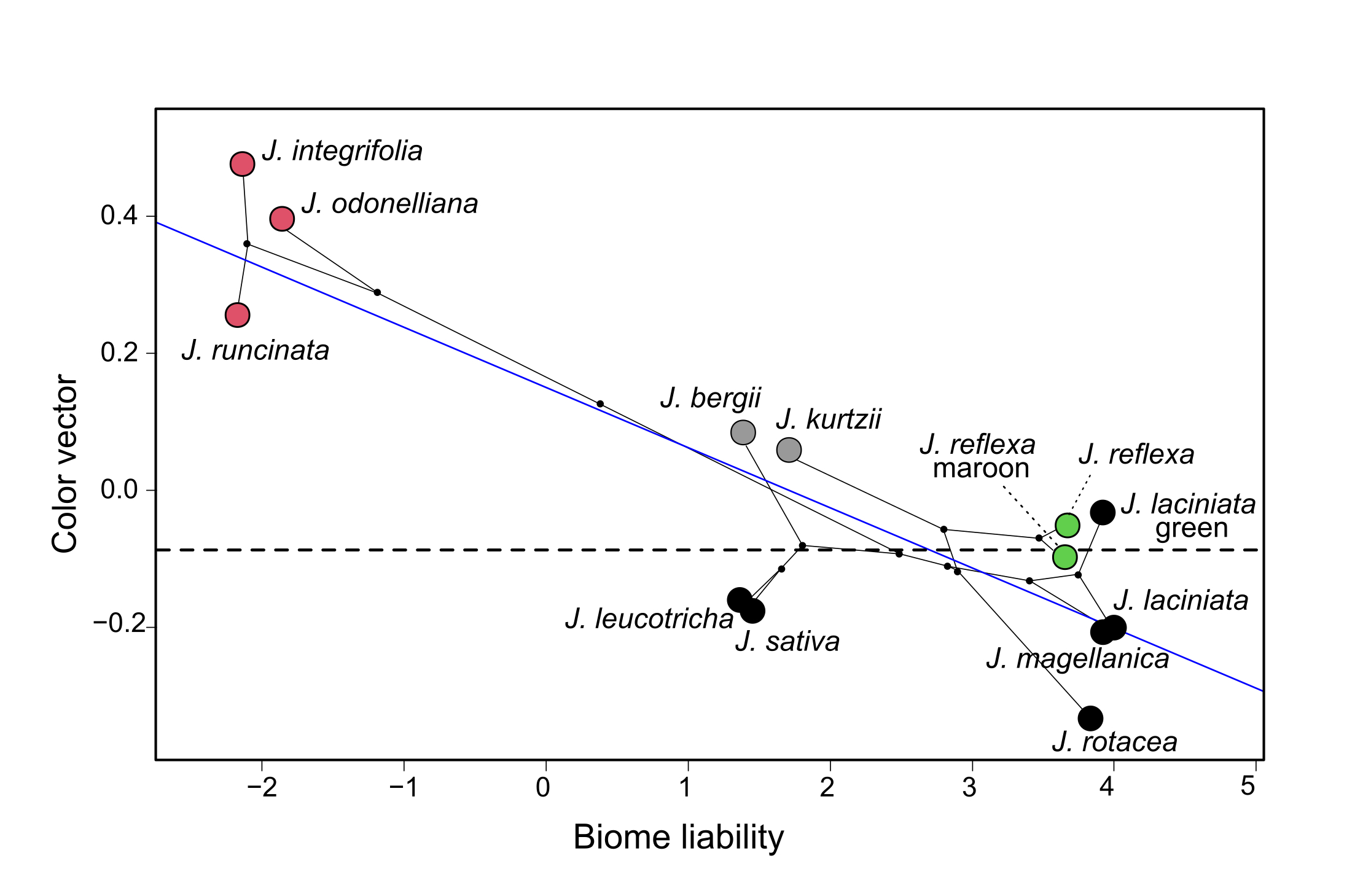

Supplement: Supplementary Figure 3 — Evolutionary transitions along the color vision axis. Phylogenetic generalized least squares correlation (PGLS) between environment occupancy and perceptual color vector of the blowfly Lucilia sp. (see text for further explanations). The dashed horizontal line (at −0.9 in the y-axis) corresponds to the center of the fly color space where all the quadrants coincide. Thus, Jaborosa species that are in the p−y− quadrant are shown above the line, and those Jaborosa species that are in the p+y+ quadrant are shown below the line. [file Image_3.JPEG]
