# Supplementary material for: Flower Diversification Across “Pollinator Climates”: Sensory Aspects of Corolla Color Evolution in the Florally Diverse South American Genus Jaborosa (Solanaceae)
Source: Front Plant Sci. 2020 Dec 7;11:601975. doi: 10.3389/fpls.2020.601975 (PMC7750315; doi:10.3389/fpls.2020.601975)
Supplement: Supplementary Table 1 — Jaborosa species studied for the corolla reflectance analyses and visual models. We provide information about pollinator observations (direct observation, ligh trapping and hawkmoths’ scales analysis) in different localities. Asterisks show confirmed pollinators. [file Table_1.docx]

|  |  | Direct observation (min) | | Nocturnal light trapping (min) |  |  |
| --- | --- | --- | --- | --- | --- | --- |
| Species | Locality | daytime | nightime |  | Flower visitors and pollinators* | Presence of moth scales |
| *J. bergii* | Villa Mercedes, San Luis | 180 | 30 |  | ants | no |
| *J. integrifolia* | Arroyito, Córdoba | 120 | 60 |  | none | yes (2 of 15 flowers) |
|  | Diamante, Entre Ríos | 90 | 180 |  | *Astylus quadrilineatus* (Melyridae) | no |
|  | Victoria, Entre Ríos | 60 | 60 |  | *Astylus quadrilineatus* (Melyridae) | no |
|  | El Fuerte, Jujuy |  |  | 480 | *Manduca tucumana* (Sphingidae)* |  |
|  | Termas de Reyes, Jujuy |  |  | 720 | *Manduca tucumana* (Sphingidae)* |  |
| *J. runcinata* | Chapicuy |  |  |  | none | yes (1 of 23 flowers) |
|  | Diamante | 90 | 180 |  | *Astylus quadrilineatus* (Melyridae) | no |
|  | Victoria | 60 | 60 |  | *Astylus quadrilineatus* (Melyridae) |  |
| *J. odonelliana* | El Jardín | 120 | 120 |  | none | yes (1 of 19 flowers) |
| *J. rotacea* | Tafí del Valle | 600 | 30 |  | *Calliphora nigribasis* (Calliphoridae)* | no |
|  |  |  |  |  | *Chrysomya albiceps* (Calliphoridae)* |  |
|  |  |  |  |  | *Cochliomyia macellaria* (Calliphoridae)* |  |
|  |  |  |  |  | *Lucilia eximia* (Calliphoridae)* |  |
|  |  |  |  |  | *Lucilia sericata* (Calliphoridae)* |  |
|  |  |  |  |  | *Myolucilia lyrcea* (Calliphoridae)* |  |
|  |  |  |  |  | *Paralucilia fulvicrura* (Calliphoridae)* |  |
|  |  |  |  |  | *Sarconesia chlorogaster* (Calliphoridae)* |  |
|  |  |  |  |  | unidentified species (Muscidae)* |  |
|  |  |  |  |  | *Oxysarcodexia varia* (Sarcophagidae)* |  |
|  |  |  |  |  | *Oxysarcodexia paulistanensis* (Sarcophagidae)* |  |
|  |  |  |  |  | unidentified Muscoidea (probably A*nthomyidae*)* |  |
|  |  |  |  |  | *Ravinia* sp. (Sarcophagidae)* |  |
|  |  |  |  |  | ants |  |
| *J. sativa* | Balcozna | 120 |  |  | *Cochliomyia macellaria* (Calliphoridae)* | no |
|  |  |  |  |  | *Myolucilia lyrcea* (Calliphoridae)* |  |
|  |  |  |  |  | *Paralucilia fulvicrura* (Calliphoridae)* |  |
|  |  |  |  |  | 5 unidentified individuals (Muscidae)* |  |
|  |  |  |  |  | 3 unidentified individuals (Sarcophagidae)* |  |
| *J. leucotricha* | El Carrizal | 120 |  |  | 23 unidentified individuals (Muscidae, Anthomyinae, Mydaeini)* |  |
|  | Potrerillos | 120 | 20 |  | *Ravinia aureopyga* (Sarcophagidae) female* |  |
| *J. laciniata* | Las Cuevas, Mendoza | 240 |  |  | *Microcerella rusca* (Sarcophagidae) male* |  |
|  |  |  |  |  | unidentified species (Calliphoridae)* |  |
|  |  |  |  |  | unidentified species (Muscidae)* |  |
|  |  |  |  |  | unidentified species (Tabanidae)* |  |
|  |  |  |  |  | unidentified species (Tachinidae)* |  |
|  | Valle Hermoso, Mendoza |  |  |  | *Chlorobrachycoma versicolor* (Calliphoridae)* |  |
|  |  |  |  |  | *Compsomyiops fulvicrura* (Calliphoridae)* |  |
|  |  |  |  |  | *Microcerella* sp. (Sarcophagidae)* |  |
|  |  |  |  |  | unidentified species (Tabanidae)* |  |
|  |  |  |  |  | unidentified species (Tachinidae)* |  |
| *J. reflexa* | Estancia La Leona, Santa Cruz | 60 |  |  | *Microcerella* sp. (Sarcophagidae) male* | no |
|  |  |  |  |  | unidentified species (Syrphidae)* |  |
|  |  |  |  |  | unidentified species (Halictidae)* |  |
| *J. magellanica* | Le Marchant, Santa Cruz | 60 |  |  | *Microcerella rusca* (Sarcophagidae) male* |  |
|  |  |  |  |  | *Microcerella spinosa* (Sarcophagidae) male* |  |
|  |  |  |  |  | *Reynoldsia* sp. (Muscidae) female* |  |
| *J. kurtzii* | Piedra del Águila, Neuquén | 70 |  |  | none |  |
|  |  |  |  |  |  |  |
