# Supplementary material for: Flower Diversification Across “Pollinator Climates”: Sensory Aspects of Corolla Color Evolution in the Florally Diverse South American Genus Jaborosa (Solanaceae)
Source: Front Plant Sci. 2020 Dec 7;11:601975. doi: 10.3389/fpls.2020.601975 (PMC7750315; doi:10.3389/fpls.2020.601975)
Supplement: Supplementary Table 2 — Environmental zones classification used in the PGLS analyses: humid lowland zone (1), dry lowland and foothill zone (2), and alpine and high latitude zone (3). [file Table_2.DOCX]

| **Environmental zone** | **Ecoregions included (Olson *et al.* 2001)** |
| --- | --- |
| 1 | Humid Espinal (> 800 mm annual rainfall) |
|  | Humid Chaco |
|  | Humid Pampas |
|  | Parana flooded savanna |
|  | Southern Andean Yungas |
|  | Southern Cone Mesopotamian savanna |
|  | Uruguayan savanna |
| 2 | Dry Chaco |
|  | Dry Espinal (< 800 mm annual rainfall) |
|  | Low Monte |
| 3 | Central Andean puna |
|  | High Monte |
|  | Patagonian steppe |
|  | Southern Andean steppe |
