# Supplementary material for: Flower Diversification Across “Pollinator Climates”: Sensory Aspects of Corolla Color Evolution in the Florally Diverse South American Genus Jaborosa (Solanaceae)
Source: Front Plant Sci. 2020 Dec 7;11:601975. doi: 10.3389/fpls.2020.601975 (PMC7750315; doi:10.3389/fpls.2020.601975)
Supplement: Supplementary Table 3 — Phylogenetic generalized least squares model among Jaborosa species between environmental occupancy and flower color expressed in hawkmoth and blowfly vision models. [file Table_3.DOCX]

|  | Color | | | |
| --- | --- | --- | --- | --- |
|  | Hawkmoth vision axis | | Blowfly vision axis | |
|  | F-value | p-value | F-value | p-value |
| Environment occupancy | 23.93 | 0.0005 | 29.05 | 0.0002 |
